# Lying with the Head High

**Published:** 1886-04

**Authors:** 


**Lying with the Head High.**—It is often a question amongst people who are unacquainted with the anatomy and physiology of man, whether lying with his head exalted or even the body, is most wholesome. Those who consult their own ease on the point argue in favor of that which they prefer. Now, although many delight in bolstering up their heads at night, and sleep soundly without injury, it is nevertheless a dangerous habit. The vessels through which blood passes from the heart to the head, are always lessened in the cavities when the head is resting in bed higher than the body; therefore, in all diseases attended with fever, the head should be pretty near on a level with the body; and people ought to accustom themselves to sleep in this position to avoid danger.

